# ED-Swin Transformer: A Cassava Disease Classification Model Integrated with UAV Images

**DOI:** 10.3390/s25082432

**Published:** 2025-04-12

**Authors:** Jing Zhang, Hao Zhou, Kunyu Liu, Yuguang Xu

**Affiliations:** 1 College of Artificial Intelligence & Computer Science, Xi’an University of Science and Technology, Xi’an 710600, China; 23208223073@stu.xust.edu.cn (H.Z.); xuyuggmw@xust.edu.cn (Y.X.); 2 School of Economics and Management, Xidian University, Xi’an 710126, China; liukunyu463@163.com

**Keywords:** UAV, image processing, plant disease, swin transformer

## Abstract

The outbreak of cassava diseases poses a serious threat to agricultural economic security and food production systems in tropical regions. Traditional manual monitoring methods are limited by efficiency bottlenecks and insufficient spatial coverage. Although low-altitude drone technology offers advantages such as high resolution and strong timeliness, it faces dual challenges in the field of disease identification, such as complex background interference and irregular disease morphology. To address these issues, this study proposes an intelligent classification method for cassava diseases based on drone imagery and an ED-Swin Transformer. Firstly, we introduced the EMAGE (Efficient Multi-Scale Attention with Grouping and Expansion) module, which integrates the global distribution features and local texture details of diseased leaves in drone imagery through a multi-scale grouped attention mechanism, effectively mitigating the interference of complex background noise on feature extraction. Secondly, the DASPP (Deformable Atrous Spatial Pyramid Pooling) module was designed to use deformable atrous convolution to adaptively match the irregular boundaries of diseased areas, enhancing the model’s robustness to morphological variations caused by angles and occlusions in low-altitude drone photography. The results show that the ED-Swin Transformer model achieved excellent performance across five evaluation metrics, with scores of 94.32%, 94.56%, 98.56%, 89.22%, and 96.52%, representing improvements of 1.28%, 2.32%, 0.38%, 3.12%, and 1.4%, respectively. These experiments demonstrate the superior performance of the ED-Swin Transformer model in cassava classification networks.

## 1. Introduction

Cassava, a vital food crop in tropical and subtropical regions worldwide, plays a crucial role in local economies and food security [1]. However, the high incidence and rapid spread of leaf diseases in cassava significantly threaten crop yield and quality. Traditional manual field inspections suffer from poor timeliness, low coverage, and strong subjectivity. Additionally, complex terrain variations, uneven soil conditions, and the singularity of the data collected using mobile cameras, along with environmental interference, further complicate disease monitoring. With the advancement of precision agriculture technologies, unmanned aerial vehicles (UAVs) have emerged as a novel solution for disease monitoring due to their efficient and flexible data acquisition capabilities [2]. Nonetheless, UAV data are susceptible to interference from lighting, weather conditions, and background noise; in addition, the high complexity of data processing poses greater challenges for disease identification [3]. Therefore, integrating UAV image acquisition with deep learning algorithms to develop precise classification methods for cassava diseases is of significant importance for effective disease control and ensuring crop yield.

As a core technological tool in precision agriculture, unmanned aerial vehicles (UAVs) are driving profound transformations in traditional agricultural production models, leveraging their efficient, flexible, and precise data acquisition capabilities. In fields such as crop growth monitoring, precise disease identification, and intelligent farmland management, UAV technology demonstrates significant application potential and value. Simultaneously, the rapid development of computer vision and deep learning technologies has provided novel technical pathways for the classification of plant leaf diseases. Currently, deep-learning-based disease classification methods are primarily divided into two categories, as follows: those based on convolutional neural networks (CNNs) [4] and those based on Transformers [5]. CNNs and their variants have achieved remarkable results in image feature extraction and classification. However, in complex agricultural environments, diseased and healthy leaves often exhibit similarities in color, texture, and shape, compounded by background interference, which limits the performance of traditional CNN methods in capturing disease features and global contextual information [6,7]. Consequently, more sophisticated models are required to effectively extract both global and local features, adapting to multi-scale visual information. Transformer models, with their powerful self-attention mechanisms, have achieved significant success in natural language processing and computer vision. Nevertheless, standard Transformers exhibit high computational complexity in image classification tasks, limiting their efficiency when processing large-scale datasets [8,9]. Swin Transformers address this by introducing a sliding window mechanism, confining self-attention computations to local regions, and thereby enhancing computational efficiency while retaining global feature extraction capabilities [10]. This makes them particularly effective in classification tasks involving complex backgrounds, though there remains room for optimization in global feature extraction.

Therefore, we propose a cassava disease classification model integrated with UAV imagery. We deployed UAVs in cassava fields to collect data on cassava disease types along fixed flight paths. After data collection, the information was transmitted to computer devices, where an improved cassava disease classification algorithm was implemented. The contributions of this paper are as follows:

(1) Leveraging the advantages of UAV data acquisition, we employed an enhanced deep learning image classification model to accurately classify comprehensive cassava disease images captured with UAVs.
(2) Firstly, we introduced the EMAGE module to integrate the global distribution characteristics and local texture details of diseased leaves in UAV imagery, effectively mitigating the interference of complex background noise on feature extraction. Additionally, we incorporated dynamic grouping and dilated convolution modules, enabling the model to adaptively adjust the number of groups and fuse multi-scale information. This addresses the issues of missing global information and insufficient detail capture in key disease regions within complex backgrounds.
(3) We designed the DASPP module, which employs deformable dilated convolutions to adaptively match the irregular boundaries of disease regions. This enhances the model’s robustness to morphological variations caused by angles and occlusions in low-altitude UAV imagery.

## 2. Related Work

### 2.1. Plant Disease Identification

Traditional approaches for identifying plant diseases often depend on manual expertise and visual inspection, which are not only laborious and time-intensive, but also prone to subjective errors. With the swift progress of artificial intelligence, deep learning techniques, especially convolutional neural networks (CNNs) and Transformer-based architectures, have shown remarkable efficacy in image classification tasks, leading to their widespread adoption in the automated detection and categorization of plant diseases [11,12]. Gaikwad et al. [13] employed deep CNNs to categorize the leaf diseases of key crops (apple, custard apple, and guava) in Karnataka, India, marking the first effort to classify these fungal leaf afflictions. Paul et al. [14] introduced a streamlined custom CNN model integrated with transfer learning frameworks, VGG-16 and VGG-19, for the classification of tomato leaf diseases. By employing data augmentation and conducting ablation studies, they refined the model, attaining peak accuracy and a recall rate of 95%. Ahad et al. [15] evaluated six CNN structures, alongside transfer learning and ensemble models, for the classification of rice diseases in Bangladesh. The ensemble model reached an optimal accuracy of 98%, demonstrating the utility of transfer learning in enhancing plant disease identification.

Although convolutional neural networks (CNNs) have demonstrated excellent performance in plant disease classification, their feature extraction capabilities are limited when handling large datasets and images with complex backgrounds, often failing to capture global information. When disease features exhibit minimal contrast with the background, CNNs struggle to detect the subtle disease characteristics, leading to a decline in classification performance. In contrast, Transformers, with their self-attention mechanisms, excel at capturing global features, enabling higher classification accuracy in complex environments. Hemalatha et al. [16] proposed a novel approach for plant disease detection and classification using a Vision Transformer (PDLC-ViT). The model incorporates same-scale, co-attention, and cross-attention mechanisms within a multi-task learning (MTL) framework, leveraging Vision Transformer (ViT) technology. Sun et al. [17] introduced the Eff-Swin model, which combines the strengths of EfficientNetV2 and Swin Transformers, effectively utilizing CNNs for local feature extraction and Transformers for global modeling. Comparative experiments have demonstrated that this enhanced model significantly improved training accuracy.

With the advancement of precision agriculture, the integration of unmanned aerial vehicles (UAVs) and computer vision technology has provided an efficient solution for plant disease monitoring. By combining aerial imagery with deep learning algorithms, the automated identification and classification of crop diseases can be achieved, significantly enhancing early warning capabilities. The Bouguettaya team [18] proposed a deep-learning- and UAV-based method for crop disease recognition, while Tetila et al. [19] demonstrated its superiority over traditional algorithms such as SIFT and SVM in soybean pest detection. Furthermore, the Jayanthy team [20] developed a real-time detection system for cotton diseases by deploying deep learning models on UAV platforms, promoting the transition toward intelligent agricultural plant protection.

### 2.2. Attention Mechanism

The attention mechanism, especially the self-attention mechanism, has achieved remarkable success in computer vision tasks [21], as it adaptively focuses on different regions of the input image, effectively extracting key features [22,23]. Currently, channel attention, spatial attention, and hybrid attention mechanisms have been widely applied in plant disease classification [24]. Bhuyan P. [25] introduced the Res4net-CBAM model, tailored for tea leaf disease diagnosis. This model integrates the Res4net framework with the CBAM module, aiming to lower computational complexity while enhancing classification performance. Zhao Y. [26] introduced SEV-Net, embedding improved channel and spatial attention modules into ResNet residual blocks to reduce information redundancy and focus on the most information-rich areas. Tang Z. et al. [27] employed ShuffleNet V1 and V2 backbone networks combined with the Squeeze-and-Excitation (SE) Block as an improved channel attention mechanism, proposing a lightweight CNN method. Gao R. [28] introduced a crop disease identification model based on dual-branch channel attention (DECA). This approach employs dual-branch 1D convolution to refine feature information and adaptively choose the relevant features, thereby improving the model’s effectiveness. Naik B. N. [29] studied the performance of different networks on a custom-built pepper leaf dataset and designed an SE-Block-based CNN (SECNN) model, significantly improving the classification results. Alirezazadeh P. [30] investigated the impact of the CBAM module on lightweight CNNs, extracting more discriminative features by highlighting the important local regions.

### 2.3. Feature Extraction

Feature extraction techniques are crucial in image processing tasks, especially for extracting multi-scale features [31,32]. Methods such as Feature Pyramid Networks (FPN) and Atrous Spatial Pyramid Pooling (ASPP) enable models to handle images with complex backgrounds more effectively [33]. Vishnoi V. K. et al. [34] reviewed the advantages and disadvantages of handcrafted and deep learning feature extraction techniques, emphasizing the critical role of feature fusion in image detection systems. Fan X. et al. [35] improved the extraction of local texture details by merging deep and traditional handcrafted features. They introduced center loss to enhance feature discriminability. Mao R. [36] proposed the DAE-Mask method, which combines DenseNet, FPN, and attention mechanisms to enhance diverse feature extraction for wheat disease detection. Yang X. [37] introduced the ISC-MRCNN method, which uses an improved FPN structure to achieve multi-scale feature fusion and more detailed information learning in plant leaf images. Dai G. et al. [38] developed the DFN-PSAN network, which integrates YOLOv5 and PSA mechanisms to successfully classify fine-grained plant diseases in natural field environments. Li Y. [39] proposed a CNN-based maize leaf disease detection method that incorporates a Coordinate Attention (CA) module and an improved SSP module, enhancing the effectiveness of feature information. Jianlou L. [40] combined the ASPP module with the SGE attention mechanism in the CSASNet model, using Leaky ReLU to optimize the training process, which improved the extraction of disease spot features. Karthik R. [41] developed a dual-branch deep learning network that integrates GSDFP and Swin Transformers, combining local and global features, and using a random attention module to capture contextual relationships, effectively classifying citrus plant diseases.

## 3. Method

We introduce the ED-Swin Transformer, an innovative deep learning architecture designed for cassava disease classification utilizing UAV-based imaging.

**Figure 1:**
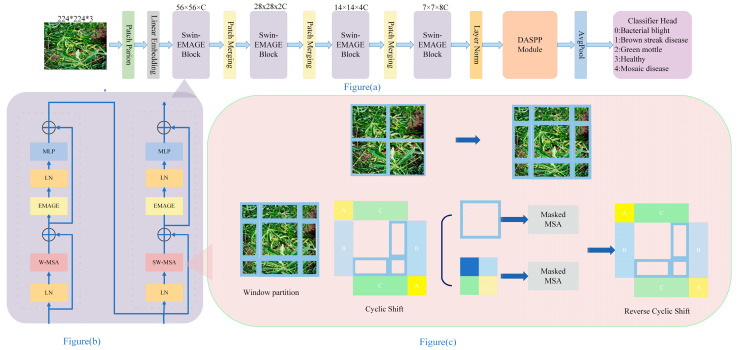
Overall architecture of the ED-Swin Transformer cassava leaf disease classification network (**a**), Swin-EMAGE Block (**b**), and shifted windows multi-head self-attention model (**c**).

The initial image with dimensions of 224 × 224 × 3 passes through the Patch Partition and Linear Embedding modules before entering the main structure of the Swin-EMAGE Block (as shown in Figure 1b). First, within the Swin-EMAGE Block, the EMAGE module is integrated into the traditional Swin Transformer architecture. By merging global and local features, this compensates for the Swin Transformer’s limitations in global feature extraction within its attention mechanism. Secondly, before the classification head, the DASPP module is applied, replacing standard convolutions and dilated convolutions with deformable convolutions. This allows the convolution kernels to dynamically adjust their sampling positions based on the input image content, better aligning with irregular disease features and capturing richer contextual information.

### 3.1. Swin Transformer

The Swin Transformer is a window-based multi-head self-attention (MSA) model. Unlike the traditional Vision Transformer (ViT), the Swin Transformer reduces computational complexity through a hierarchical structure and windowing mechanism, enabling it to process higher-resolution images more efficiently. The core of the Swin Transformer lies in its hierarchical architecture and sliding window mechanism. Each layer consists of the following key sub-modules: Patch Partition, Linear Embedding, Swin Transformer Block (including W-MSA and SW-MSA), and Patch Merging [42]. The key formulas for this module are as follows:

(1) $$\overset{\wedge }{Z}=W-MSA(LN(Z^{L}))+Z^{L-1}$$

(2) $$Z^{L}=MLP(LN(\overset{\wedge }{Z^{L}}))+\overset{\wedge }{Z^{L}}$$

(3) $$Z^{L+1}=SW-MAS(LN(Z^{L}))+Z^{L}$$

(4) $$Z^{L+1}=MLP(LN(\overset{\wedge }{Z^{L+1}}))+\overset{\wedge }{Z^{L+1}}$$

### 3.2. EMAGE

The Efficient Multi-Scale Attention with Grouping and Expansion (EMAGE) module enhances the integration of global and local features, compensating for the Swin Transformer’s limitations in global feature extraction. The EMAGE module strengthens the model’s ability to capture multi-scale contextual information, thereby improving its focus on both global and local features.

**Figure 2:**
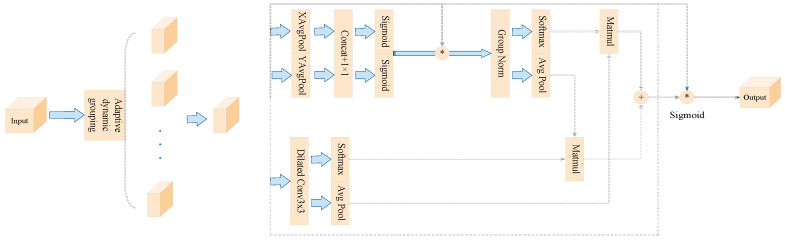
The overall architecture of the EMAGE module.

The EMAGE module combines adaptive dynamic grouping, pooling, normalization, and convolution to improve multi-scale feature extraction, as illustrated in Figure 2. Dynamic grouping adapts to input data characteristics, organizing the features based on their properties, as outlined in Equation (5). The module employs two 1D average pooling operations along channel directions for encoding. The encoded channel features are merged along the height dimension of the image, using a shared 1 × 1 convolution kernel [43]. The result is processed through a Sigmoid activation function, splitting it into two vectors along the H and W axes. These vectors undergo 2D binomial transformation and linear convolution before being reweighted for adaptive feature selection, yielding the 1 × 1 branch output. For the 3 × 3 branch, a single 3 × 3 dilated convolution extracts multi-scale features, producing the branch’s output. The cross-spatial learning module uses 2D global average pooling to encode global information from the 1 × 1 branch output, activated by Softmax. This output is then multiplied with the 3 × 3 branch output to create the first spatial attention map [44]. Similarly, the 3 × 3 branch output undergoes 2D global average pooling for global encoding, activated by Softmax, and is multiplied with the group-normalized 1 × 1 branch output. This produces the second spatial attention map, which preserves precise spatial information through 2D average pooling, as detailed in Equation (6).

(5) $$g=\max(1,\;\min(factor,\;\frac{c}{\min c})$$

(6) $$Z_{c}=\frac{1}{H\times W}\sum_{j}^{H}\sum_{i}^{W}x_{c}(i,\;j)$$

The factor represents molecular group base counts, where min c and c denote the minimum and input feature channels, respectively. After dynamic grouping, g indicates grouped channels, while H and W are feature map dimensions. $x_{c}$ represents the channel-wise feature tensor. The spatial attention map employs aggregated Sigmoid-activated functions to select the reweighted adaptive features for a global context. EMAGE combines 3 *×* 3 dilated convolution and 1 *×* 1 convolutions to extract multi-scale contextual information, enhancing pixel-level attention on deep features. Parallel convolution kernels address both short- and long-term dependencies through cross-space learning. Integrating EMAGE into an ED-Swin Transformer creates a Swin-EMAGE Block that synergistically combines local and global features, overcoming the Swin Transformer’s global extraction limitations while boosting network discriminative power. Our method shows superior performance in cassava leaf disease identification, demonstrating enhanced robustness and generalization across diverse environments and disease manifestations.

### 3.3. DASPP

**Figure 3:**
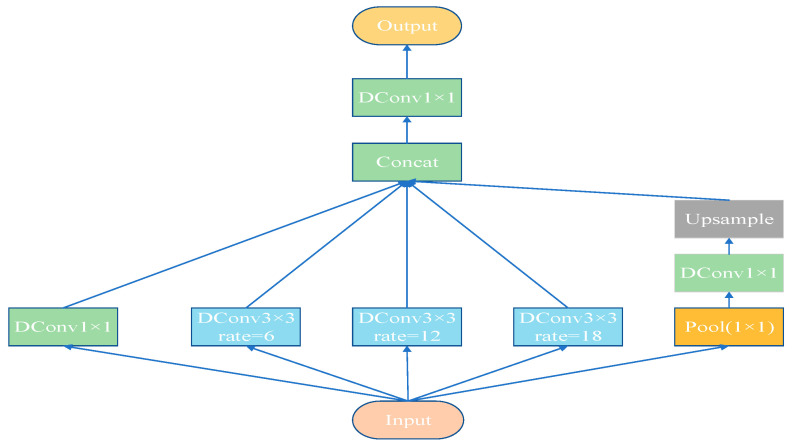
The overall architecture of the DASPP module.

Deformable Atrous Spatial Pyramid Pooling (DASPP) is an enhanced feature extraction module that utilizes multiple parallel deformable atrous convolution layers to perform multi-scale sampling on the input image at different dilation rates. As shown in Figure 3, compared to traditional Atrous Spatial Pyramid Pooling (ASPP), DASPP replaces standard convolutions and dilated convolutions with deformable convolutions. By combining the dilation operation of atrous convolutions with the dynamic properties of deformable convolutions, the convolutional kernels can not only expand the receptive field, but also dynamically adjust the sampling positions. This enables more effective capture of multi-scale and complex-shaped features.

By using different dilation rates (such as 1, 6, 12, and 18), the DASPP module is able to generate multiple sets of feature maps. Larger dilation rates help to capture broader contextual information, while smaller dilation rates preserve the finer details of the image. After being processed by dynamic deformable convolutions, these feature maps are concatenated along the channel dimension, forming a multi-scale feature representation. In this way, DASPP enables the simultaneous acquisition of both local details and global contextual information on the same feature map, enhancing the model’s ability to represent features across different scales.

Due to the irregular and complex shapes of cassava leaf disease lesions, the model highly depends on feature information from different receptive fields. In this study, we integrated the DASPP module before the classification head to overcome the limitations of traditional methods in classifying overlapping or irregular objects under different receptive fields. By combining deformable convolutions with the ASPP module, DASPP significantly enhances the model’s ability to classify overlapping leaves. The fused feature maps are then fed into the classification head, further improving classification accuracy.

## 4. Results

### 4.1. Data Preparation

**Figure 4:**
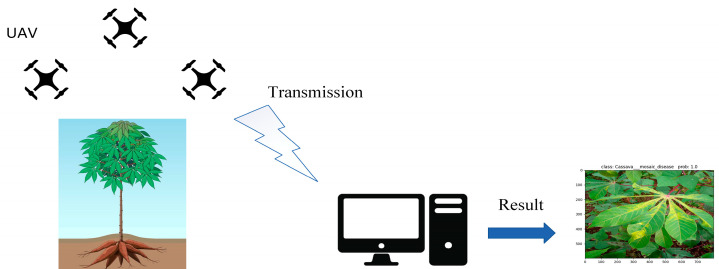
Data collection and transmission process.

UAV-based image acquisition offers exceptional safety and flexibility, enabling adaptation to complex and variable environmental conditions with minimal impact from environmental changes. The short image acquisition cycle significantly enhances work efficiency while substantially reducing labor costs. Figure 4 illustrates the complete process of obtaining cassava disease images using UAVs. First, the high-definition camera mounted on the UAV captures images of cassava diseases and transmits them in real time to computer devices via a wireless network. Subsequently, the image classification algorithm deployed on the device categorizes the cassava diseases in the images, and the classification results are finally visualized on the device.

**Figure 5:**
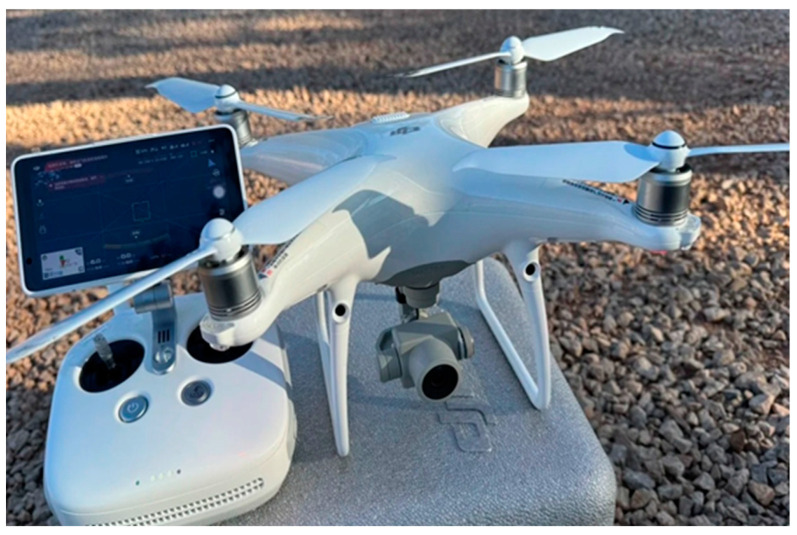
DJI Phantom 4 Pro V2.0 drone.

In this study, we developed a custom cassava disease dataset for model training, with field data collection conducted in Jiangzhou District, Chongzuo City, Guangxi Zhuang Autonomous Region. The equipment employed a DJI Phantom 4 Pro V2.0 unmanned aerial vehicle (UAV) equipped with a 20-megapixel CMOS sensor (1-inch format; 24 mm fixed focal length; f/5.6 aperture; ISO 200), as shown in Figure 5, combined with ground-level mobile camera imaging. The images were captured at approximately 5 m from the cassava plants to document various disease manifestations. All collected images underwent professional manual annotation and classification. To enhance dataset diversity and model generalization capability, we integrated our field-collected data with publicly available datasets and applied multiple augmentation techniques, including rotation, mirroring, Gaussian noise addition, and color jittering. The final comprehensive dataset comprised 54,353 images across the following five distinct categories: Bacterial blight (5435 images), Brown streak disease (10,945 images), Green mottle (11,930 images), healthy plants (12,885 images), and mosaic disease (13,158 images). The dataset was strategically partitioned into training and testing sets at an 8:2 ratio to ensure robust model evaluation, with healthy plant specimens representing the largest category, followed closely by mosaic disease cases.

### 4.2. Experimental Parameter Settings and Metric Evaluation

The equipment and parameters used in the experiments are summarized below. The operating system was Windows, equipped with an NVIDIA GeForce RTX 2080 Ti graphics card. The CPU was an Intel (R) Core (TM) i9-9820X CPU @ 3.30 GHz. The training and compilation were conducted using Python 3.7.2, and the deep learning framework utilized was PyTorch 1.10.0. During the training process, we used the AdamW optimizer with a batch size of 8. The weight decay was set to 5 × 10^−2^,, the minimum learning rate was set to 1 × 10^−4^, and the training was conducted for a total of 50 epochs.

We selected accuracy, precision, recall, specificity, and F1 score to evaluate our classification model. These metrics can be calculated using the following formulas:

(7) $$accuracy=\frac{TP+TN}{N}$$

(8) $$precision=\frac{TN}{FP+FP}$$

(9) $$recall=\frac{TP}{TP+FN}$$

(10) $$Specificity=\frac{TP}{FP+TN}$$

(11) $$F1=\frac{2\times precision\times recall}{precision+recall}$$

where TP refers to the number of true positives, FP refers to the number of false positives, and FN refers to the number of false negatives.

### 4.3. The Ablation Experiments on the ED-Swin Transformer

We conducted ablation experiments on different attention mechanisms, as shown in Table 1. The baseline Swin Transformer achieved 93.04% accuracy, 92.24% precision, 98.18% recall, 86.10% specificity, and a 95.12% F1 score, with 48.3M parameters and 4.5G FLOPs. By integrating the EMAGE module with dynamic grouping and dilated convolutions, the model demonstrated superior performance, achieving 93.76% accuracy, 93.66% precision, 98.44% recall, 88.20% specificity, and a 95.99% F1 score, while maintaining efficiency at 48.3M parameters and 8.5G FLOPs. This enhancement effectively extracts global contextual information, improving the fusion of global and local features in the Swin Transformer. The results confirm that EMAGE mitigates global information loss and enhances detail capture in critical diseased areas under complex backgrounds.

To assess the contribution of the DASPP module, we performed comprehensive ablation studies, with the results summarized in Table 2. The Swin Transformer augmented with DASPP consistently outperformed competing approaches across all evaluation metrics—including accuracy, precision, recall, specificity, and F1 score—while remaining computationally efficient (49.3M parameters, 9.3G FLOPs). These findings indicate that the deformable convolutions in DASPP excel at capturing diverse morphological features of cassava leaf diseases compared to both FPN and conventional ASPP methods.

This paper proposes the ED-Swin Transformer network, an efficient architecture with 49.3 M parameters and 9.3 G FLOPs, integrating EMAGE and DASPP modules for enhanced cassava disease classification. The ablation studies in Table 3 demonstrate consistent performance gains, as follows: while the baseline achieves 93.04% accuracy, adding the lightweight EMAGE module improves the results to 93.76% without increasing the computational costs. The DASPP variant further elevates the accuracy to 94.08% with minimal overhead, and our full ED-Swin Transformer achieves state-of-the-art performance at 94.32% accuracy and 96.52% F1-score. The model balances efficiency and capability, excelling in complex disease pattern recognition—the specificity improves by 2.1% with EMAGE and 3.22% for the complete system over the baseline, despite its compact design.

To comprehensively evaluate the model’s performance, this study conducted a systematic ablation analysis based on the internationally recognized plant disease benchmark dataset, PlantVillage. As shown in Table 4, all comparative models demonstrated excellent classification performance. Among them, the baseline Swin Transformer model achieved a benchmark accuracy of 97.03%, while the proposed ED-Swin Transformer model significantly outperformed the comparative models across the key metrics, including accuracy at 98.43%, precision at 98.39%, and recall at 97.91%. The model not only exhibited outstanding performance on the standard dataset, but also demonstrated superior generalization capability when tested on a self-constructed field dataset. These results robustly validate the universality and practical value of the ED-Swin Transformer model in plant disease classification tasks.

### 4.4. Effectiveness Evaluation of ED-Swin Transformer

**Figure 6:**
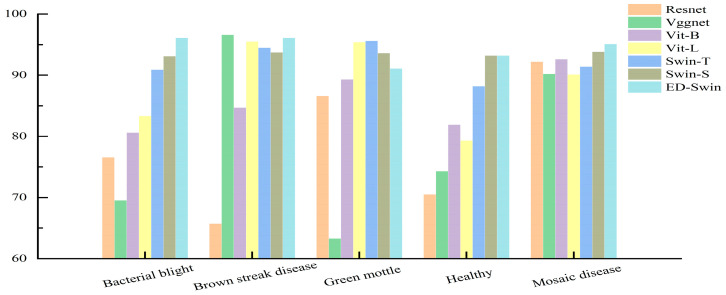
Performance of different models on specific cassava diseases.

We conducted experiments to evaluate the recognition performance of different models on specific types of cassava diseases, and the results are shown in Table 5 and Figure 6. Our model achieved excellent accuracy for all five types of cassava diseases, with rates of 96.1%, 96.1%, 91.1%, 93.25%, and 95.1%, respectively. In particular, the model performed exceptionally well on Bacterial blight, health plants, and mosaic disease. On the other hand, the accuracy of Resnet and Vggnet was relatively lower, possibly due to the limitations of traditional CNN models in capturing spatial information. This limitation becomes more apparent with large-scale datasets that include multiple scales, perspectives, and complex backgrounds. CNNs may not be powerful enough to effectively separate features from different categories. In contrast, Transformer-based models can adjust feature extraction more flexibly and better accommodate the needs of large-scale datasets. Compared to traditional convolutional neural networks, Vit models showed improvements across all five evaluation metrics. However, when compared to the Swin Transformer, Vit still fell slightly behind. The Swin Transformer uses a window-based attention mechanism to capture global information, resulting in robust accuracy across different disease types. Specifically, Swin-T achieved accuracy rates of 90.9%, 94.5%, 95.6%, 88.2%, and 91.4%, while Swin-S reached 93.1%, 93.7%, 93.6%, 93.2%, and 93.8%. It is worth noting that the images in the dataset were captured in natural environments, which inevitably introduce some noise. Moreover, different cassava diseases exhibit different characteristics, and the sensitivity of various models to diseased regions may vary. Therefore, the accuracy in identifying different diseases may differ between models.

To validate the effectiveness of our approach, we compared the ED-Swin Transformer with leading classification models, including CNNs like Resnet and Vggnet, as well as Vision Transformers such as Vit-B, Vit-L, Swin-T, and Swin-S. As shown in Table 6, the ED-Swin Transformer achieved superior performance with 94.32% accuracy, 94.56% precision, 98.56% recall, 89.22% specificity, and 96.52% F1 score. The traditional CNNs demonstrated significantly lower results, with Resnet and Vggnet achieving only 78.32% and 78.18% accuracy, respectively, along with specificity below 60.69%, highlighting their limited discriminative ability on negative samples. While Vit-B and Vit-L improved upon CNNs with 85.82% and 88.72% accuracy, they still fell short of the Swin-based models. Swin-T and Swin-S delivered a stronger performance at 92.12% and 93.04% accuracy, yet our ED-Swin Transformer surpassed them in all metrics. Notably, the ED-Swin Transformer maintained computational efficiency, matching Swin-S in model size at 49.3M parameters and 9.3G FLOPs. This evaluation confirms our architecture’s superiority in balancing accuracy, robustness, and efficiency

**Figure 7:**
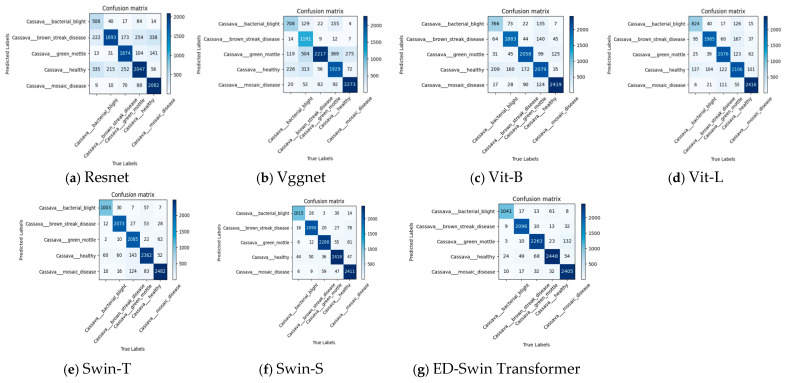
Confusion matrices of different models. (**a**) Resnet, (**b**) Vggnet, (**c**) Vit-B, (**d**) Vit-L, (**e**) Swin-T, (**f**) Swin-S, and (**g**) ED-Swin Transformer.

In this study, seven different models were used to extract disease lesions from the surface of cassava leaves. These models include Resnet, Vggnet, Vit-B, Vit-L, Swin-T, Swin-S, and the ED-Swin Transformer. Compared to other methods, the ED-Swin Transformer model captured more significant features from the extracted lesions. Figure 7 presents the confusion matrices obtained from these seven models.

### 4.5. Visualized Results of Different Models in Cassava Leaf Disease Classification

**Figure 8:**
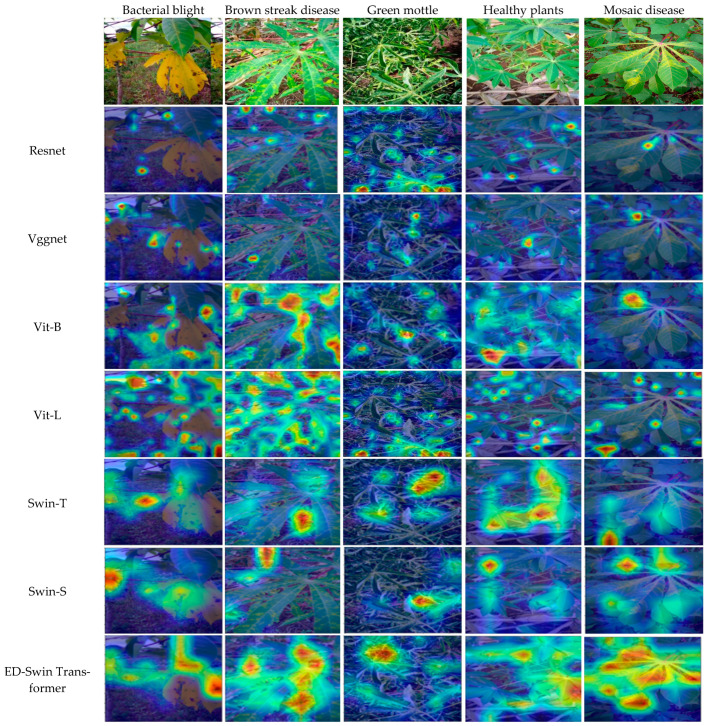
The Grad-CAM visualization results for different models.

Different lesion extraction algorithms employ varying attention mechanisms, which may introduce errors during image analysis. In this context, accurately identifying the region of interest (ROI) becomes crucial. To provide more intuitive insights, we utilized Grad-CAM—a widely adopted visualization tool—to generate heatmaps highlighting model attention. Figure 8 presents the Grad-CAM visualization results across the different models. The Class Activation Mapping (CAM) technique generates attention heatmaps by performing weighted fusion on the feature maps from the final convolutional layer. These heatmaps intuitively reveal the model’s decision-making process, particularly for the critical regions that contribute most to its final classification judgment. In the task of leaf disease diagnosis, CAM visualizations effectively identify the key visual features that the model relies on to distinguish between healthy and diseased leaves. While the focus points vary across the different models, the Vision Transformer (ViT) and Swin Transformer networks generally exhibit superior feature extraction capabilities. Notably, the proposed ED-Swin Transformer model captures a greater number of discriminative regions across different lesions and demonstrates enhanced ability in identifying diverse disease features. This indicates that the ED-Swin Transformer-based cassava disease classification network effectively mitigates key challenges, including global information loss, insufficient localization of diseased regions, and irregular lesion manifestations in a complex background.

## 5. Conclusions

This paper addresses the challenges of complex background interference, global information loss, insufficient detail capture in key disease regions, and irregular lesion morphology in cassava disease classification using UAV imagery. We propose an intelligent cassava leaf disease classification network based on ED-Swin Transformers. By integrating the efficient and flexible data acquisition capabilities of UAVs with the feature extraction strengths of deep learning, this network achieves precise disease classification in complex scenarios.

Firstly, the EMAGE module was designed to integrate the global distribution characteristics and local texture details of diseased leaves in UAV imagery through a multi-scale grouped attention mechanism. This effectively mitigates the interference of complex background noise on feature learning in low-altitude photography. Secondly, the DASPP module was introduced, employing deformable dilated convolutions to adaptively match the irregular lesion boundaries in UAV imagery caused by shooting angles, occlusions, or disease spread. This enhances the model’s robustness to morphological variation features.

The experimental results demonstrate that the proposed ED-Swin Transformer model, based on UAV imagery, effectively validates the synergistic role of hybrid attention mechanisms and deformable convolutions in agricultural UAV image analysis tasks. Particularly, it highlights the unique adaptability and significant advantages of artificial intelligence technology in complex agricultural scenarios. This study not only provides an innovative solution for UAV-driven intelligent agricultural disease monitoring, but also offers a methodological framework with strong scalability, which can be widely applied to large-scale disease diagnosis for various crops. It holds substantial practical significance for the construction of food security systems in tropical regions. However, this work has certain limitations. The model’s performance may be affected by extreme environmental conditions not fully represented in the current dataset, such as heavy occlusion or highly variable lighting. Furthermore, while the method shows promising results on tropical crops, its generalizability to other agricultural regions requires further validation. Building on the current research outcomes, future studies will focus on exploring lightweight deployment solutions for the model, aiming to promote its practical application in edge-based UAV agricultural IoT systems. This effort will provide robust technical support for the intelligent transformation of precision agriculture.

## Figures and Tables

**Table 1:** The ablation experiments on the ED-Swin Transformer.

	Accuracy/%	Precision /%	Recall/%	Specificity/%	F1 Score/%	Params/M	FLOPs /G
Swin Transformer	93.04	92.24	98.18	86.10	95.12	48.3	8.5
Swin Transformer + CBAM	93.30	93.32	98.32	87.83	95.75	48.4	8.6
Swin Transformer + EMA	93.74	93.00	98.26	87.42	95.55	48.3	8.5
Swin Transformer + EMAGE	93.76	93.66	98.44	88.20	95.99	48.3	8.5

**Table 2:** The ablation experiments on the ED-Swin Transformer.

	Accuracy/%	Precision /%	Recall /%	Specificity /%	F1 Score/%	Params /M	FLOPs /G
Swin Transformer	93.04	92.24	98.18	86.10	95.12	48.3	8.5
Swin Transformer + FPN	93.24	93.32	98.28	87.28	95.74	49.8	8.9
Swin Transformer + ASPP	93.42	93.40	98.48	88.30	95.87	49.1	8.2
Swin Transformer + DASPP	94.08	93.76	98.48	88.92	96.06	49.3	9.3

**Table 3:** The ablation experiments of the ED-Swin Transformer in the private dataset.

	Accuracy /%	Precision /%	Recall /%	Specificity /%	F1 Score/%	Params /M	FLOPs /G
Swin Transformer	93.04	92.24	98.18	86.10	95.12	48.3	8.5
Swin Transformer + EMAGE	93.76	93.66	98.44	88.20	95.99	48.3	8.5
Swin Transformer + DASPP	94.08	93.76	98.48	88.92	96.06	49.3	9.3
ED-Swin Transformer	94.32	94.56	98.56	89.22	96.52	49.3	9.3

**Table 4:** The ablation experiments of the ED-Swin Transformer on PlantVillage.

	Accuracy /%	Precision /%	Recall /%	Specificity /%	F1 Score/%
Swin Transformer	97.03	96.14	95.8	96.19	96.00
Swin Transformer + EMAGE	97.42	96.63	96.38	96.81	96.50
Swin Transformer + DASPP	97.91	97.41	97.23	97.59	97.32
ED-Swin Transformer	98.43	98.39	97.91	98.95	98.14

**Table 5:** Comparison of accuracy for five disease categories across different models.

Accuracy/%	Bacterial Blight	Brown Streak Disease	Green Mottle	Healthy Plants	Mosaic Disease
Resnet	76.6	65.7	86.6	70.5	92.2
Vggnet	69.5	96.6	63.3	74.3	90.2
Vit-B	80.6	84.7	89.3	81.9	92.6
Vit-L	83.3	95.5	95.4	79.3	90.1
Swin-T	90.9	94.5	95.6	88.2	91.4
Swin-S	93.1	93.7	93.6	93.2	93.8
ED-Swin Transformer	96.1	96.1	91.1	93.2	95.1

**Table 6:** Comparative experiments of the ED-Swin Transformer model.

	Accuracy/%	Precision/%	Recall/%	Specificity/%	F1 Score/%	Params/M	FLOPs /G
Resnet	78.32	74.04	94.24	60.69	82.93	25.5	3.8
Vggnet	78.18	74.74	94.02	60.60	83.28	138	15.4
Vit-B	85.82	85.40	96.62	75.14	90.66	86	17.6
Vit-L	88.72	87.84	97.04	78.65	92.21	304	122
Swin-T	92.12	92.08	97.98	85.32	94.94	28	4.5
Swin-S	93.04	92.24	98.18	86.10	95.12	48	8.5
ED-Swin Transformer	94.32	94.56	98.56	89.22	96.52	49.3	9.3

## Data Availability

The data presented in this study are available upon request from the corresponding author. Due to project confidentiality, the dataset cannot be disclosed at this time.
